# Resting Sinus Heart Rate and First Degree AV block: Modifiable Risk Predictors or Epiphenomena?

**Published:** 2009-11-01

**Authors:** Rakesh Gopinathannair, Brian Olshansky

**Affiliations:** Division of Cardiovascular Medicine, University of Iowa Hospital, Iowa City, IA

**Keywords:** Sinus heart rate, first degree AV block, risk predictors, cardiovascular disease

## Abstract

Simple and cost-effective tools that identify patients at increased risk for adverse cardiovascular events are actively sought. High resting sinus heart rate and first degree AV block are easily recognized and commonly encountered findings in a cardiology practice. A growing body of epidemiological and clinical evidence has been shown them to be independent predictors of all-cause and cardiovascular mortality, both in the general population and in patients with structural heart disease. This paper reviews the important role of heart rate and first degree AV block in predicting cardiovascular outcomes, examines the pathophysiological mechanisms underlying this increased risk, and discusses the effectiveness of available therapies to favorably modify these risk factors.

## Introduction

There is considerable interest in identifying asymptomatic individuals in the general population who are at risk for cardiovascular events. Multiple risk predictors are available and more are being recognized. In this report, we focus on the prognostic significance of resting sinus heart rate (HR) and first degree AV block, both commonly encountered and easily measured parameters in day-to-day cardiology practice.

## Resting heart rate

Heart rate is perhaps the simplest and most easily measured vital sign in cardiovascular medicine.  Arbitrary definitions have been established and followed for both bradycardia and tachycardia over the years. Gradations of HR within the normal range (60-100 bpm) and their relationship to cardiovascular disease and outcomes have not been carefully evaluated until recently.

Over the past decade, a number of large, longitudinal epidemiological studies have shown that high resting sinus HRs are independently associated with adverse cardiovascular outcomes in the general population as well as in patients with hypertension, coronary artery disease, and heart failure [1-3]. High sinus HRs have been linked to hypertension, progression of coronary artery disease, myocardial infarction, cardiomyopathy, and sudden death [2,4-6]. Even small increments in HR within the 'normal' range (60-100 bpm) as well as increases in HR over time were also associated with a worse prognosis [3,6,7]. Jouven et al studied 5713 asymptomatic men without any structural heart disease. Subjects underwent baseline treadmill exercise testing and were followed over a 23-year period. Those with a resting HR > 75 bpm, a HR increase with exercise < 89 bpm, or a HR decrease in recovery < 25 bpm had a significantly higher risk of sudden death when adjusted for potential confounding variables [6]. In a post-hoc analysis of the LIFE (Losartan Intervention For End point reduction in hypertension) study, Okin et al showed that higher in-treatment sinus HRs independently predicted an increased risk of atrial fibrillation in patients with hypertension and left ventricular hypertrophy treated with atenolol or losartan  [7].

In patients with coronary artery disease and left ventricular ejection fraction < 40%, a baseline resting HR> 70 bpm predicted a 34% increased risk of cardiovascular death, 53% increased risk of heart failure hospitalizations, and a 38% increased  risk of hospitalization for myocardial infarction.  Above 65 bpm, every 5 bpm increase in baseline HR resulted in an 8% increase in cardiovascular death and a 16% increase in heart failure hospitalizations  [3].

The predictive value of resting sinus HR also extends to females at risk for cardiovascular disease. A cohort of ~123,000 postmenopausal women followed prospectively over an 8-year period showed that higher resting sinus HRs, measured at baseline by palpating the radial pulse, independently predicted myocardial infarction and coronary death.  The association was the strongest in women between 50-64 years of age [8].

Although simple to measure, it is important to identify the correct way to measure true baseline HR. Previous studies have employed different types of HR measurement including resting HR from radial pulse palpation or from baseline electrocardiogram, mean HR over 24 hours, HR variability, sleep HR and recovery HR after treadmill exercise testing [3,6,8,9]. Whether a particular measure is better than another in reliably assessing the prognostic significance of HR needs to be explored in future studies.

A convincing relationship thus exists between higher resting sinus HR and adverse cardiovascular outcomes. The underlying mechanisms, however, remain less clear. Direct effects could involve increase in myocardial oxygen demand, reduction in coronary perfusion, increase in pulsatile shear stress resulting in endothelial dysfunction, acceleration of atherosclerosis and plaque rupture, and increase in cardiac norepinephrine synthesis [10]. On the other hand, elevated resting HR may reflect adverse changes in autonomic balance. In patients with heart failure, higher HRs could be a compensatory neurohormonal mechanism early on which when sustained, accelerates disease progression, triggers unfavorable remodeling, and thus negatively affects prognosis [11]. Alternatively, elevated resting sinus HR could be just an early marker of the severity of underlying illness/substrate.

Heart rate control in patients with pure tachycardia-induced cardiomyopathy results in almost complete recovery of heart failure and left ventricular systolic function [12]. Beta-blocker mediated reduction in heart rate has been considered partially responsible for the beneficial effects in heart failure and coronary artery disease [13,14]. Patients with atrial fibrillation managed with a rate control strategy have similar outcomes to those patients in whom rhythm control is employed [15,16]. However, what is optimal rate control is often debated [17] and a post-hoc comparison of a lenient (<100 bpm) versus a stricter rate control strategy (≤ 80 bpm) showed similar outcomes [18]. The minimal threshold HR as well as the change in HR beyond which cardiac risk increases remains unclear. However, based on current data, it would be reasonable to assume that persistent resting sinus HR >70 bpm would be of concern.

Whether higher resting sinus HR is a truly modifiable risk factor is yet to be determined. The available data so far is not promising. The multicenter, randomized BEAUTIFUL (morBidity mortality EvAlUaTion of the If inhibitor ivabradine in patients with coronary disease and left ventricULar dysfunction) trial compared the effects of ivabradine, a selective If current blocker that reduces HR independent of autonomic effects, to placebo in patients with coronary artery disease and left ventricular dysfunction. The study showed that although ivabradine reduced resting HR when compared to placebo, it did not affect the primary composite endpoint of cardiovascular death, hospitalizations for heart failure and/or acute myocardial infarction. However, ivabradine did reduce hospital admission for fatal and non-fatal myocardial infarctions and coronary revascularizations [19]. These results may reflect ivabradine-mediated attenuation of the direct effects of higher HRs on myocardial oxygen demand and coronary perfusion. In the LIFE study, a higher in-treatment sinus HR still predicted an increased risk of atrial fibrillation irrespective of the patient being in the atenolol or losartan arm [7]. A meta-analysis of 22 randomized controlled trials that evaluated beta-blocker use in hypertensive patients showed that beta-blocker mediated HR reduction increased the risk for cardiac events and death [20].

Although optimal HR for any particular individual may vary depending on demographics and comorbidities, currently available data would suggest that every effort should be made to keep the resting HR below 70 bpm. Otherwise healthy individuals with resting sinus HRs above this range should be counselled on regular physical exercise and weight reduction. Medications that increase resting heart rate should be avoided. Use of Ivabradine should be considered in patients with ischemic cardiomyopathy with resting HRs >70 bpm to reduce adverse coronary outcomes, especially if there is limiting angina [21]. At present, except in select patient populations, we do not have conclusive evidence that pharmacological therapy aimed at reducing sinus HR will improve survival.

## First degree AV block

First degree AV block, defined as a PR interval >200 msec, is commonly encountered in cardiology practice.  First degree AV block reflects slowing of atrioventricular conduction and although the AV node is the most common site, conduction delay can occur anywhere from the atrium to the infra-hisian conduction system. More than one site of conduction delay is often seen [22,23].

Many cardiovascular conditions as well as medications contribute to first degree AV block, but it can also be seen in a small percentage of healthy individuals [24,25]. Traditionally considered a benign form of conduction disturbance, marked first degree AV block (PR ≥ 300 msec), however, can lead to symptoms similar to those seen in pacemaker syndrome [26,29].  Physiologically, marked first degree AV block can lead to decreased cardiac output that may not be well tolerated, especially in patients with pre-existing left ventricular dysfunction. Lack of atrial contribution to left ventricular filling, shortening of the left ventricular diastolic filling time and development of diastolic mitral regurgitation all contribute to a reduction in left ventricular dp/dt max and cardiac output [27]. This situation can be compensated in some patients where there is marked inter-atrial conduction delay, thus allowing for an appropriate left-sided AV interval and resultant adequate left ventricular filling.

Pathophysiology as noted in patients with markedly long PR interval is also seen in AAI(R) pacing with a long PR interval as well as VVI pacing with VA conduction. In patients with markedly prolonged PR interval, dual chamber and biventricular pacing can be complicated by functional atrial undersensing. In this situation, high atrial rates predispose the atrial event to consistently fall in the PVARP thereby preventing initiation of an AV delay. This can result in AV dyssynchrony, loss of ventricular resynchronization, and symptoms consistent with a pacemaker-like syndrome [27].

The results of the recent Managed Ventricular Pacing versus VVI 40 Pacing (MVP) trial provided insights into the prognostic significance of first degree AV block in a heart failure population. The trial randomized 1031 ICD patients with sinus rhythm and no pacing indications to VVI pacing at 40 bpm (n=513) or atrial pacing at 60 bpm (MVP; n=518). Patients were followed for a mean of 25.8±10 months. The primary endpoint was a composite of time to death, heart failure hospitalization, and heart failure-related urgent care. First degree AV block was present in 15% of the study population and a PR interval >230 msec was associated with a 2.79 times increased risk of the primary composite endpoint. Furthermore, in those patients assigned to atrial pacing, every 10 msec increase in PR interval >184 msec was associated with a 12% risk of death or heart failure [30]. In patients randomized to the Cardiac Resynchronization Therapy (CRT) arm in the Multicenter InSync Randomized Clinical Evaluation (MIRACLE) and MIRACLE-ICD trials, absence of first degree AV block was associated with a favorable response to CRT (defined as an improvement of ≥1 New York Heart Association class from baseline to the 6-month follow-up) [31]. Markedly prolonged PR intervals in the CRT population, in addition to reducing percentage of biventricular pacing, could also result in loss of 'concealed resynchronization' whereby the hemodynamic benefit resulting from fusion of electrical wave fronts from the right bundle branch and the left ventricular electrode stimulation is lost [27]. It could also be that first degree AV block reflects a sicker patient group.

There are limited data on whether reduction in PR interval by pacing affects outcomes. Small, uncontrolled studies show that symptomatic patients with a PR interval ≥ 300 msec and normal left ventricular function improve with dual chamber pacing and this is considered a Class IIa indication for permanent pacing [26,32]. When comparing AAI and DDD modes of pacing in patients with sick sinus syndrome, normal ejection fraction and long PR interval, Iliev et al found that those with an AV interval <270 msec had a higher aortic velocity time integral (which reflects stroke volume and cardiac output) with AAI pacing whereas those patients with AV interval >270 msec had a higher aortic velocity time integral with DDD pacing. They also noted that at a pacing rate of 90 bpm, DDD was superior to AAI pacing [33].

On the other hand, those with left ventricular dysfunction and marked first degree AV block present a dilemma as these patients would be committed to high percentage of right ventricular pacing and its attendant risks. This was demonstrated in the Dual Chamber and VVI Implantable Defibrillator (DAVID) trial where patients with first degree AV block who were assigned to the DDDR arm had similar outcomes with DDDR pacing when compared to patients without first degree AV block [34]. These results suggest that in dual chamber systems, increased frequency of right ventricular pacing would offset the potential benefit of AV synchrony achieved by shortening the AV delay. Determining this risk-benefit ratio in a patient with left ventricular dysfunction may be difficult as acute hemodynamic and symptomatic improvement with dual chamber pacing may not guarantee long-term benefit [27,28].

One could argue that these patients would benefit from CRT and a recent sub-analysis from the CARE-HF trial lends support to this hypothesis. Predictive value of baseline and 3-month electrocardiographic variables were evaluated in heart failure patients randomized to medical therapy plus CRT (n=409) versus medical therapy alone (n=404). The primary endpoint was a composite of death from any cause and unplanned hospitalization for management of a major cardiovascular event. At baseline, PR interval >200 msec was present in 31% of patients in the CRT arm and 21% in the medical therapy arm. The study showed that prolonged PR interval at baseline and at 3 months predicted an unfavorable outcome and those patients who developed shorter PR intervals during the first 3 months after randomization had more favorable outcomes. Shortening of PR interval with CRT partially explained the effects of CRT on outcomes [35]. What PR interval is too long, what is optimal, and how best to optimize the AV delay for maximal hemodynamic benefit needs further exploration. Prospective, randomized controlled trials are being planned to address this issue and will hopefully provide us with answers in the near future.

The long-term prognostic significance of asymptomatic first degree AV block in ambulatory individuals without structural heart disease was recently evaluated in a prospective cohort study of 7575 ambulatory men and women as part of the Framingham study [36]. The mean age of the cohort was 47 years (54% women). 124 subjects had first degree AV block and predefined endpoints (all-cause mortality, atrial fibrillation, and pacemaker implantation) were assessed over a follow-up period of 20 years.  Subjects with first degree AV block (PR interval >200 msec), when compared to those who did not, had a higher incidence (per 10000 person years) of all-cause mortality, atrial fibrillation, and pacemaker implantation. First degree AV block was associated with a 2-fold adjusted risk of atrial fibrillation and a 1.4 adjusted risk of all-cause mortality. When considered as a continuous variable, each 20 msec increase in PR interval was associated with an 11% increase in atrial fibrillation, 22% increase in pacemaker implantation, and 8% increase in all-cause mortality.  This risk persisted after adjusting for interim development of structural heart disease and use of AV nodal blockers [36].

This study thus shows that asymptomatic first degree AV block in the absence of overt structural heart disease has prognostic significance and could be a marker of development of an arrhythmogenic substrate. It may be a sign of 'physiological aging' [36]. Exact underlying mechanisms and appropriate follow up for these patients is yet to be determined.  Whether early identification and intervention by pacing can lead to amelioration of this risk needs further study.

## Conclusions

Slower resting HRs are associated with longevity. Higher resting sinus HR and presence of first degree AV block are simple, yet potent measures to predict adverse cardiovascular outcomes including all-cause mortality, cardiovascular death, atrial fibrillation, coronary events and heart failure hospitalizations. There is no question that these commonly encountered signs deserve closer attention.  In terms of resting sinus HRs, it may be time to redefine current definitions of tachycardia [37-38]. The mechanisms behind higher HR and first degree AV block mediated adverse cardiac outcomes, appropriate follow up of at-risk individuals and the ability to modify these risk factors needs further evaluation.
